# Supplementary material for: Synthesis, Formulation and Characterization of Immunotherapeutic Glycosylated Dendrimer/cGAMP Complexes for CD206 Targeted Delivery to M2 Macrophages in Cold Tumors
Source: Pharmaceutics. 2022 Sep 6;14(9):1883. doi: 10.3390/pharmaceutics14091883 (PMC9503622; doi:10.3390/pharmaceutics14091883)
Supplement: Supplementary file 1 [file pharmaceutics-14-01883-s001.zip › pharmaceutics-1838722 - supplementary.pdf]

pharmaceutics

Synthesis, Formulation and Characterization of Immunotherapeutic Glycosylated Dendrimer/cGAMP Complexes for CD206 Targeted Delivery to M2 Macrophages in Cold Tumors

Supplementary data:

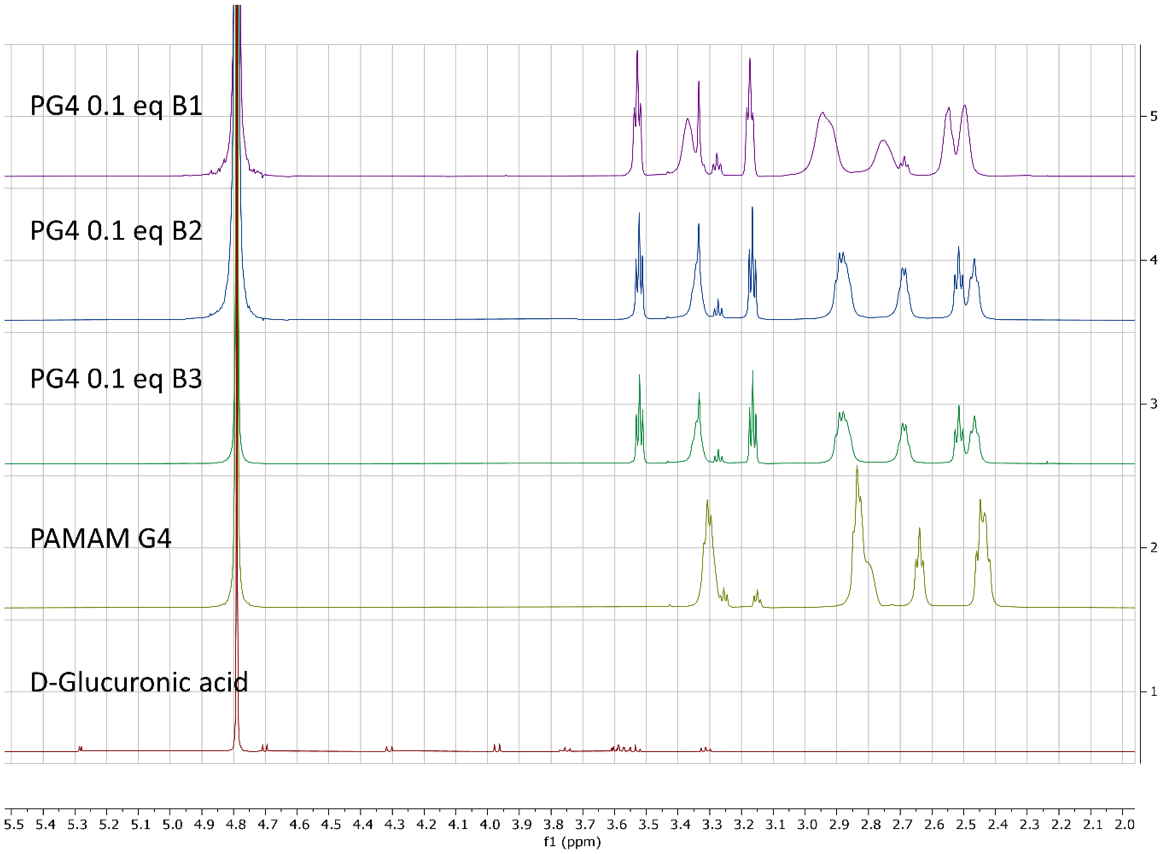

(a)

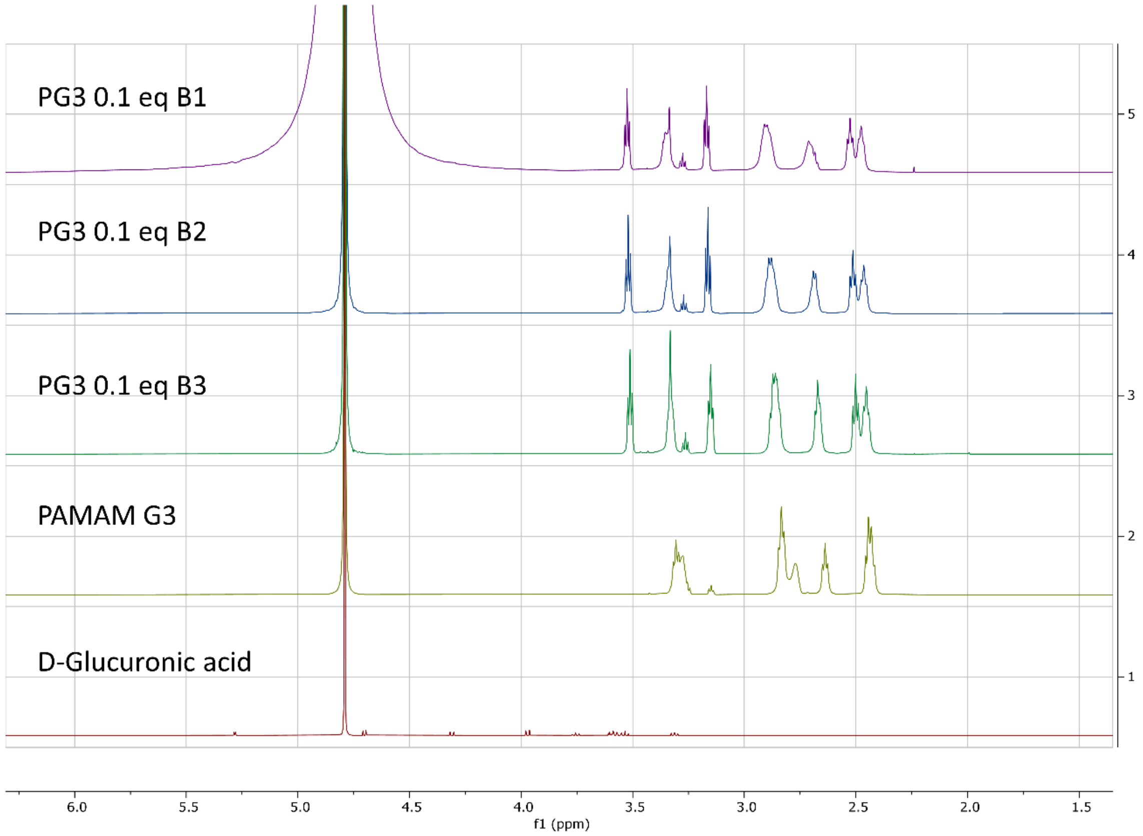
PG3 0.1 eq B1
PG3 0.1 eq B2
PG3 0.1 eq B3
PAMAM G3
D-Glucuronic acid
f1 (ppm)

(b)

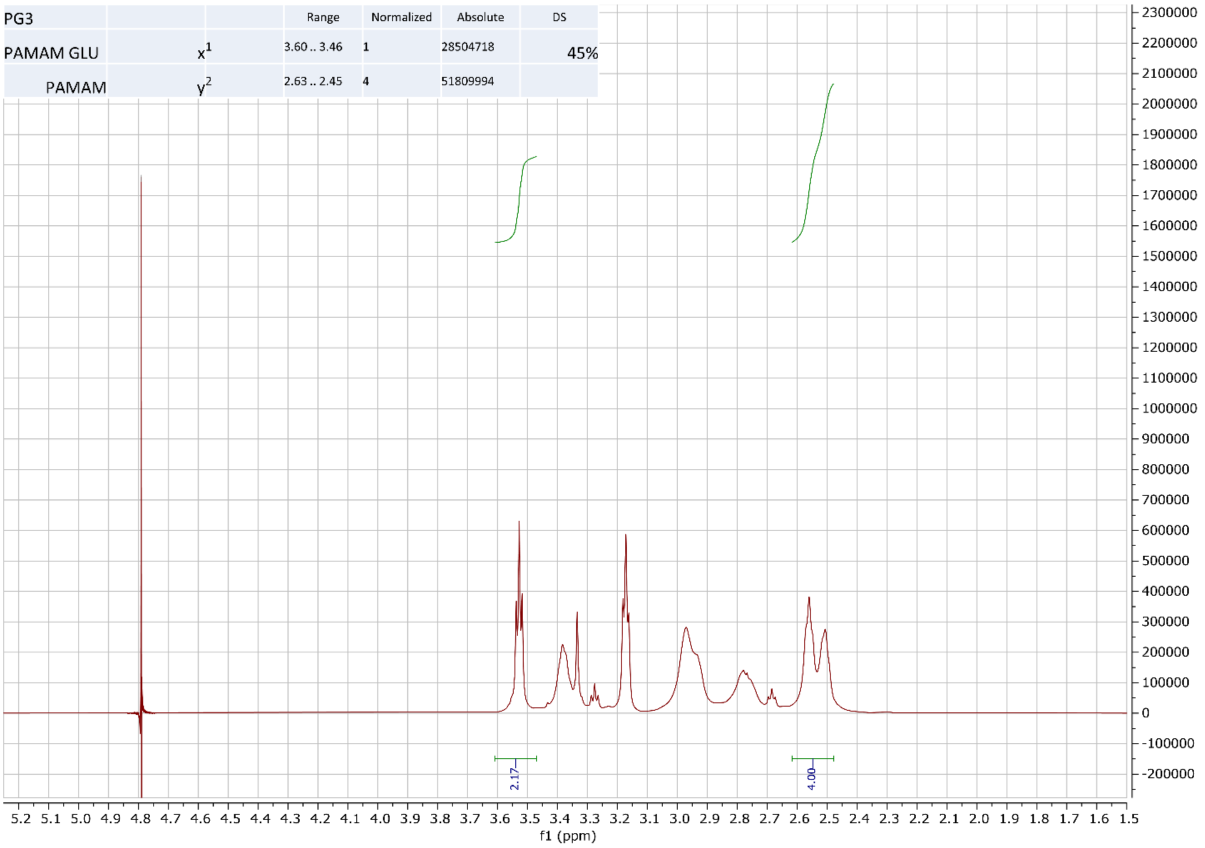
PG3
Range
Normalized
Absolute
DS
PAMAM GLU
x1
3.60 .. 3.46
1
28504718
45%
PAMAM
y2
2.63 .. 2.45
4
51809994
2.17
4.00
f1 (ppm)

(c)

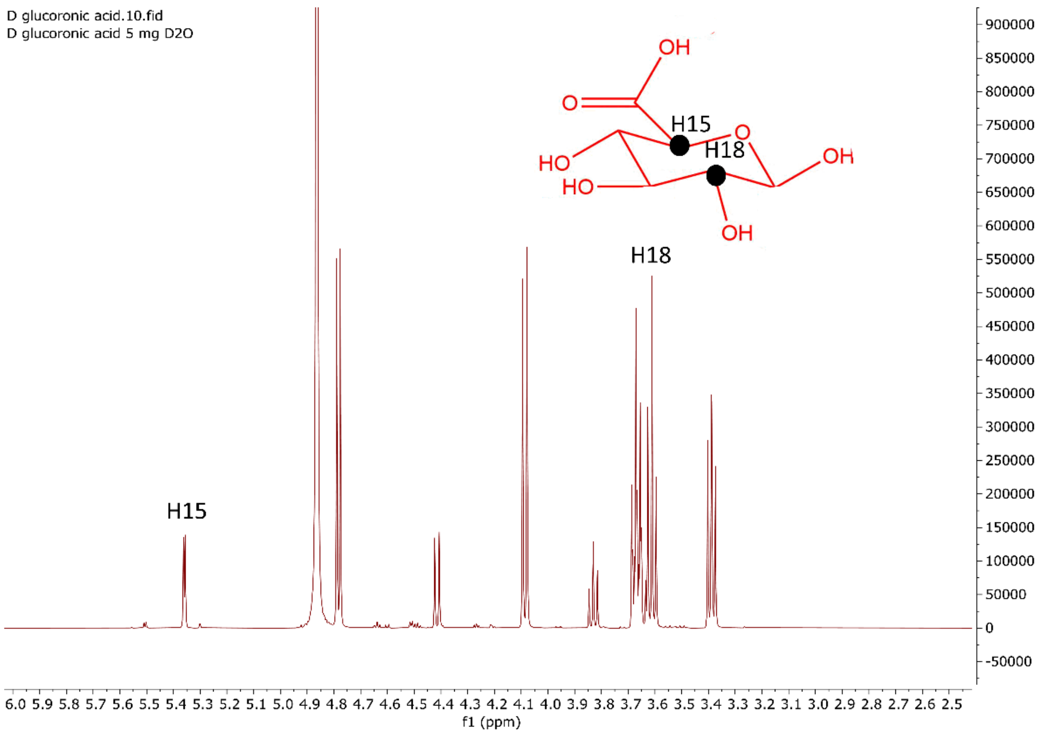

(d)

Figure S1. NMR graphs and integration of (**a**) PG4 0.1 eq batches overlap (**b**) PG3 0.1 eq batches overlap and (**c**) PG3 0.1 eq integration example (**d**) D-Glucuronic acid assignment.

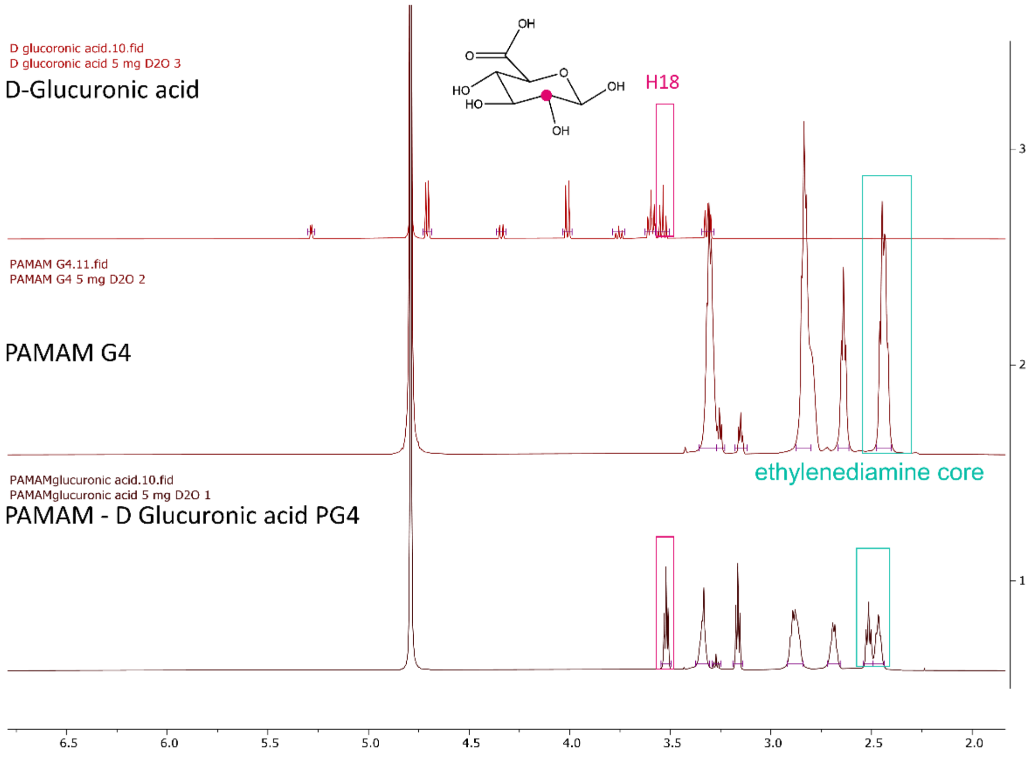

Figure S2. PAMAM G4 grafted with D-Glucuronic acid (PG4 0.1 and 0.3 eq) synthesis and characterization: NMR peak representation.

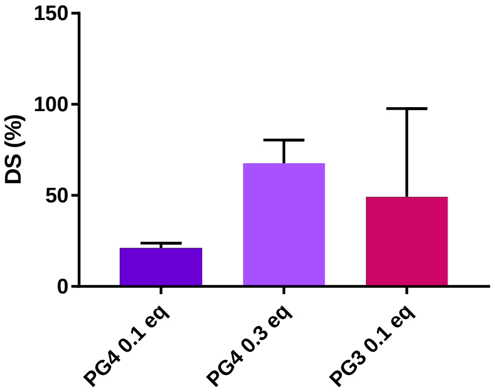

Figure S3. PAMAM amidation by Glucuronic acid; Gravimetry - DS was calculated by gravimetry and the results were 68 ± 13% for PG4 0.3, 21 ± 3% for PG4 0.1 and 49 ± 48% for PG3 0.1 eq. Results are presented as a mean ± SD. n = 3.

Table S1. The residual moisture levels after lyophilization were determined by the Karl–Fisher method.

Products	*Residual Moisture Level [%]*
PAMAM G4	*8.2–5.6*
PAMAM G3	*7.8*
PG4 0.1 eq	*3.2*
PG4 0.3 eq	*6.8*
PG3 0.1 eq	*4.6*

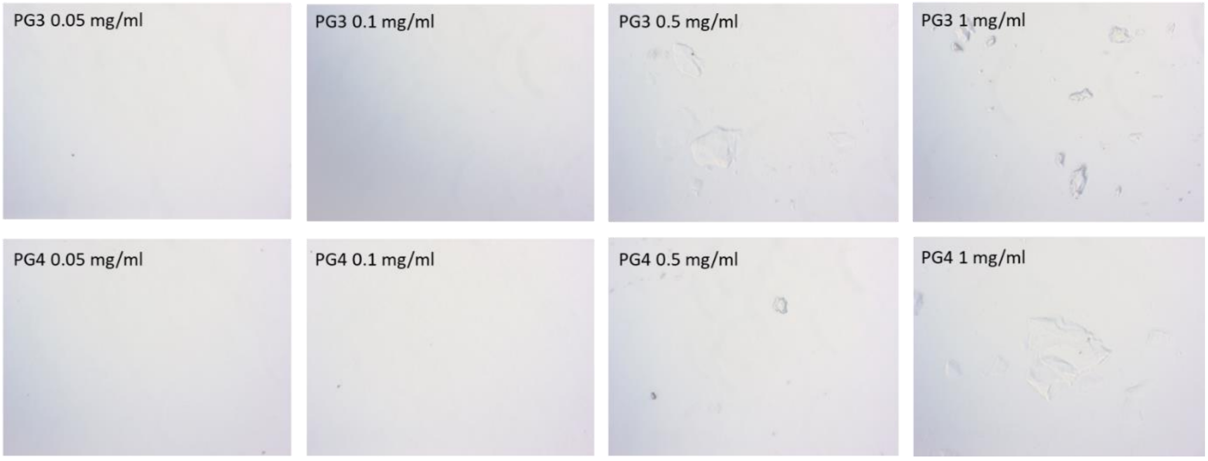

(a)

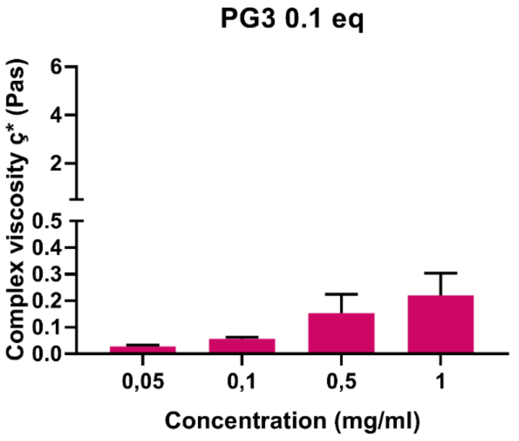

(b)

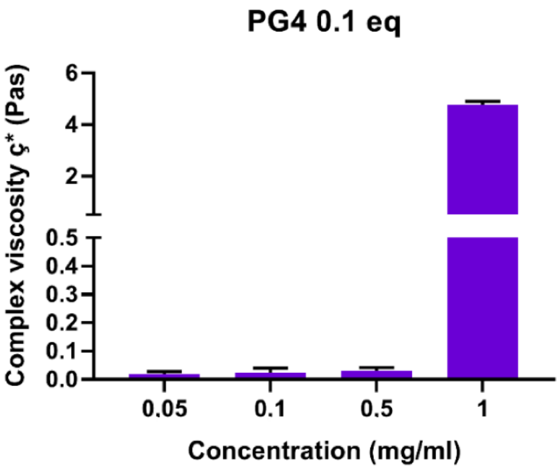

(c)

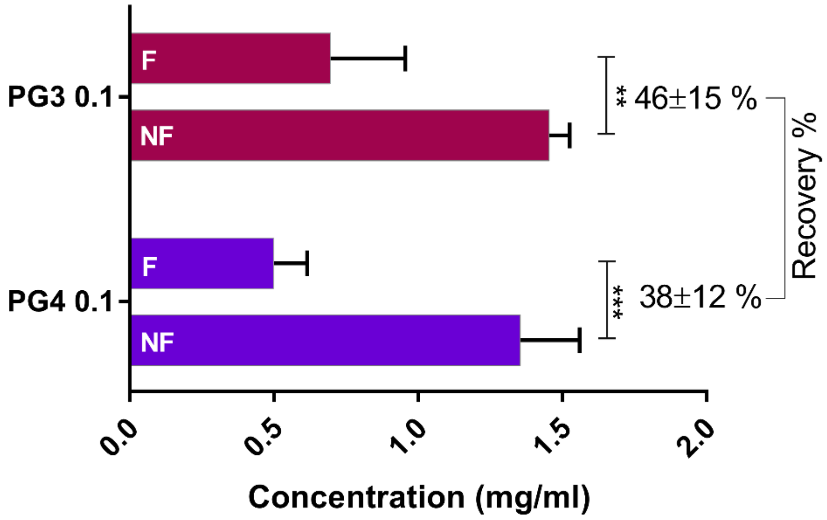

(d)

Figure S4. It was observed that both PG3 and PG4 tend to form gels at concentrations higher than 0.5 mg/ml (**a**) Light microscopy - images confirmed the presence of gel domains with the formation of a network due to the aggregation and the interactions of modified dendrimers at 0.5 mg/ ml and 1 mg/ml for both polymers. (**b**,**c**) Rheological behaviors, expressed as complex viscosity at 0.05, 0.1 and 0.5 mg/ml (i.e., 0.02 Pa·s) and more than 20 times higher at 1 mg/ml. PG3 0.1 eq showed different behavior with an increase of viscosity from 0.02 to 0.2 Pa·s linked too to concentration (**d**) comparison on PG recovery at 1 mg/ml, non-filtered (NF) and filtered over 0.45 μm filter (F) after lyophilization (optical microscopy, 10X). Recovery (%) was calculated as a weight difference between F and NF Concentration of F sample was considered as gelation concentration and further work was performed below this concentration. Results are presented as a mean ± SD. n = 3. $^{**}p < 0.01$; $^{***} p < 0.001$.

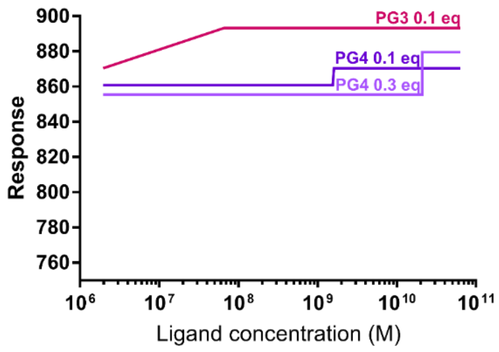

(a)

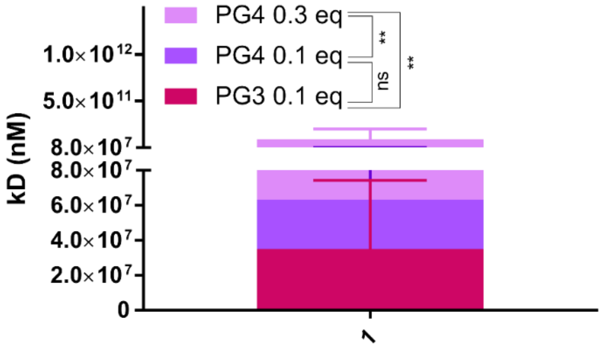

(b)

Figure S5. (**a**) Affinity of glu-modified dendrimers for CD206: MST graphical and (**b**) bar comparison for PG3 0.1 eq, PG4 0.1 eq and 0.3 eq affinity towards CD206. Results are presented as a mean ± SD. n = 3. ** $p < 0.01$.

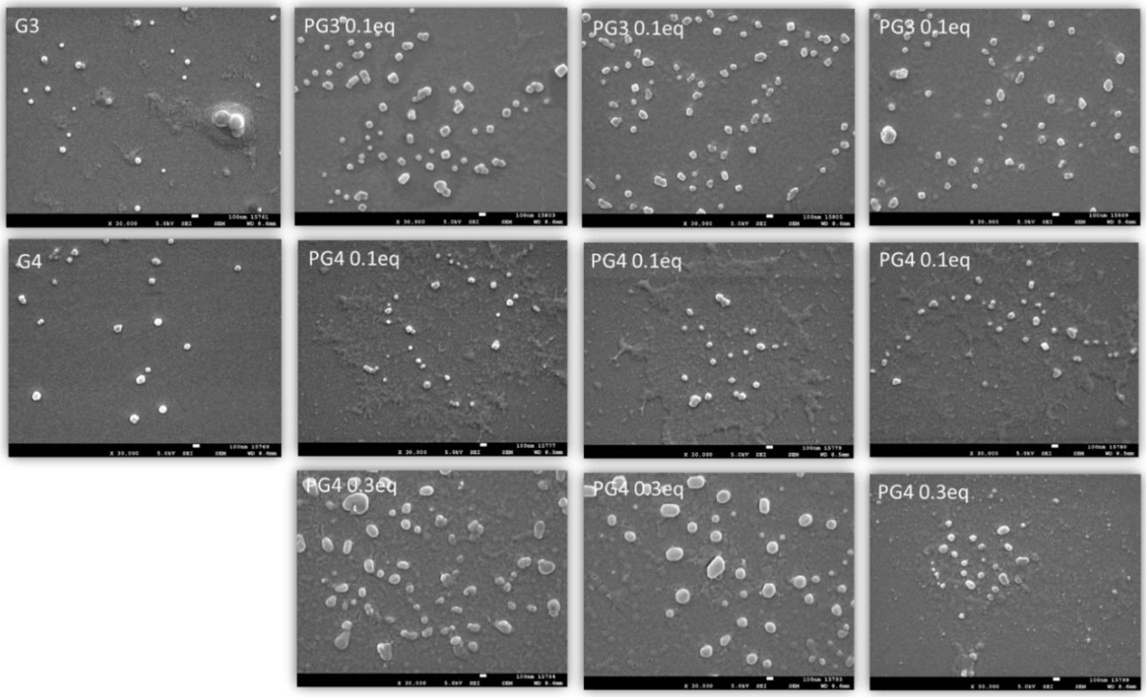

(a)

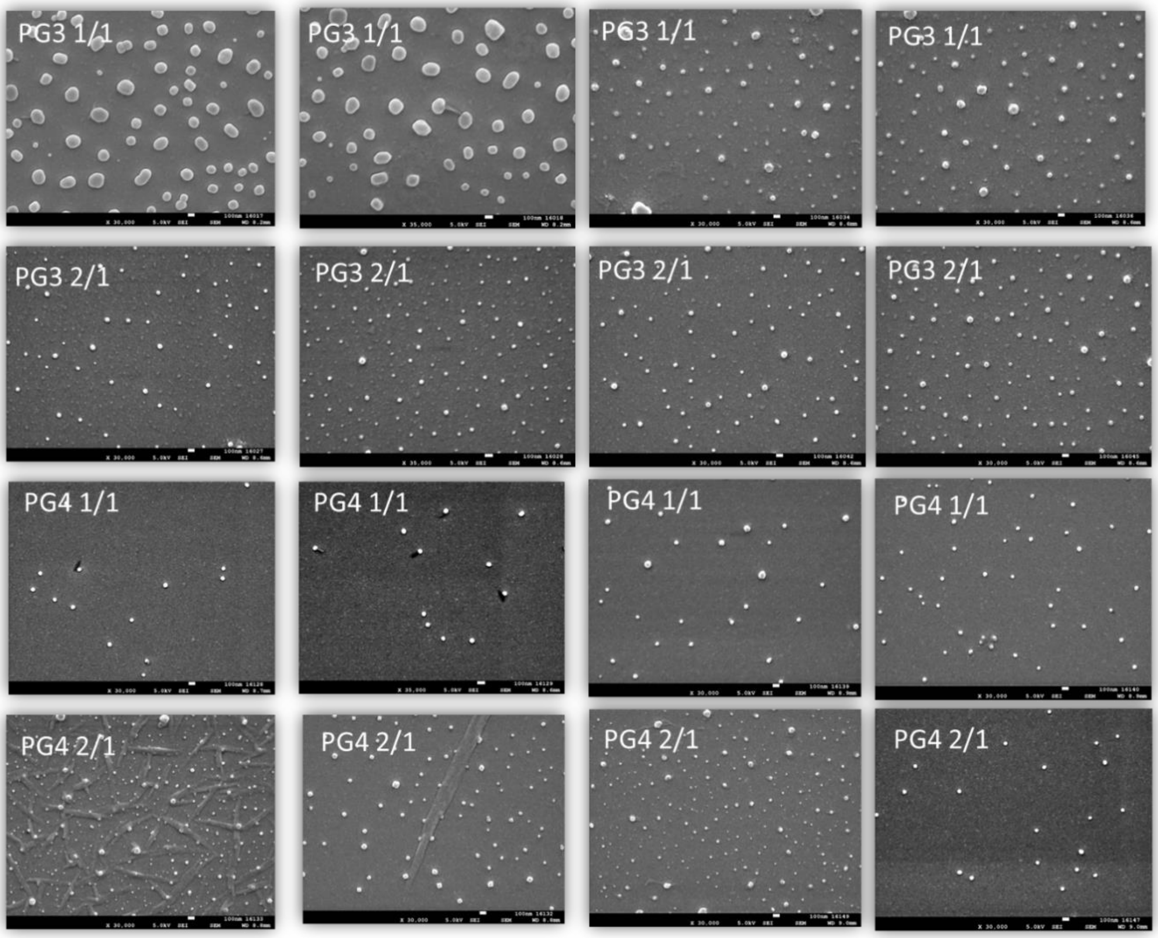

(b)

Figure S6. (**a**) SEM of comparison of three batches of PG3 0.1 eq, PG4 0.1 and 0.3 eq (**b**) NCs of 1/1 and 2/1 0.1 eq PG3 and PG4. Magnification 30 000 X, cf 100 nm bar.

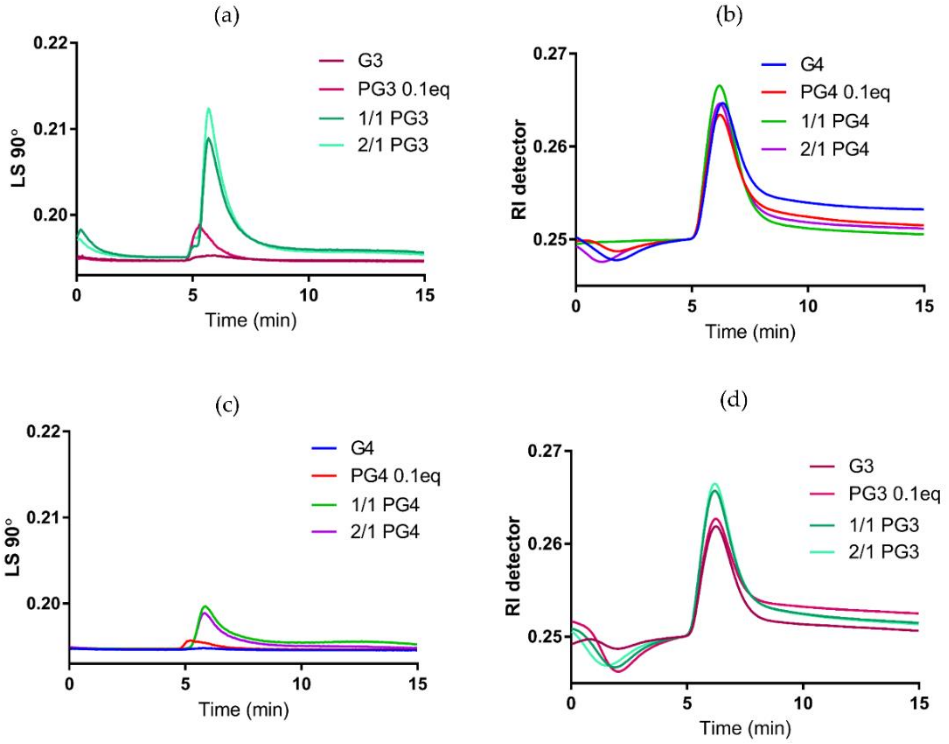

Figure S7. (**a**,**c**) AF4-MALS signal on different NCs (**b**,**d**) AF4-RI on different NCs.

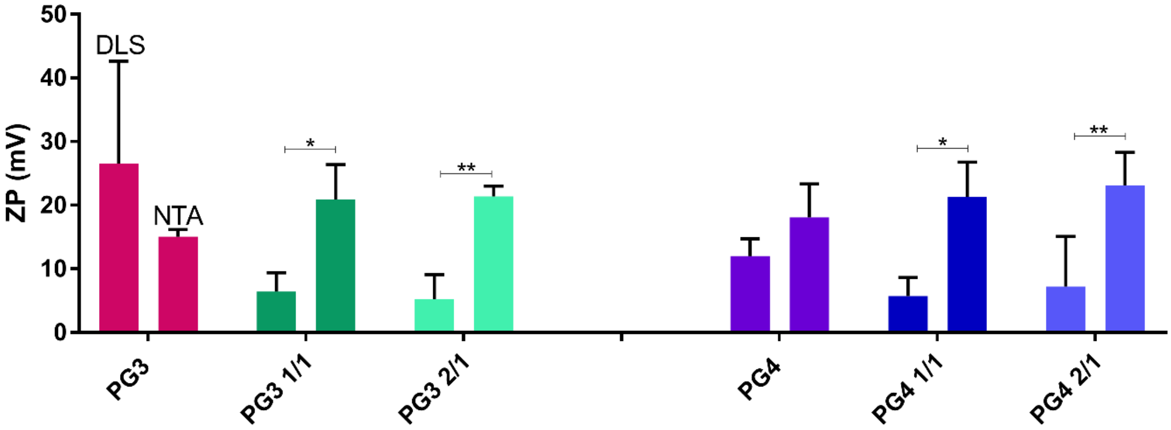

Figure S8. Comparison of zeta potential as measured by two technique (DLS and NTA) for control compounds PG3 and PG4 and their corresponding NCs Results are presented as a mean ± SD. n = 3. * $p < 0.05$; ** $p < 0.01$.

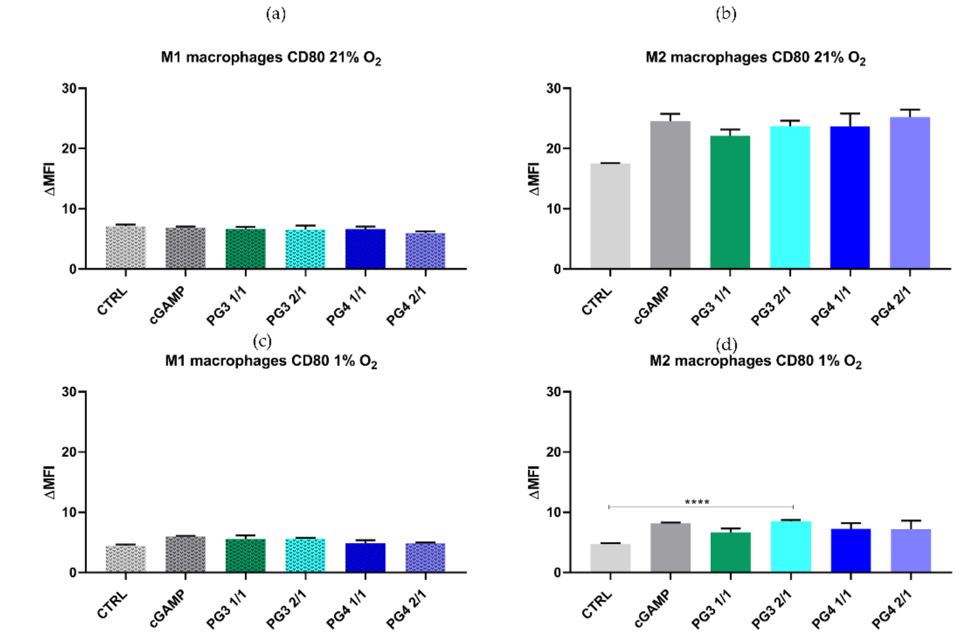

Figure S9. Surface expression of costimulatory molecule CD80 on murine BMDM cells. Polarized M1 and M2 BMDMs were stimulated in vitro with corresponding PAMAM NCs (PG3 1/1, PG3 2/1, PG4 1/1 or PG4 2/1), cGAMP (25 µg/condition) or with media under normoxic (**a**,**b**) or hypoxic (**c**,**d**) conditions for 24 h. The cells were stained with fluorophore-conjugated antibodies against the CD86 markers and analyzed by flow cytometry. Error bars are SD of three independent experiments. **** $p < 0.0001$.

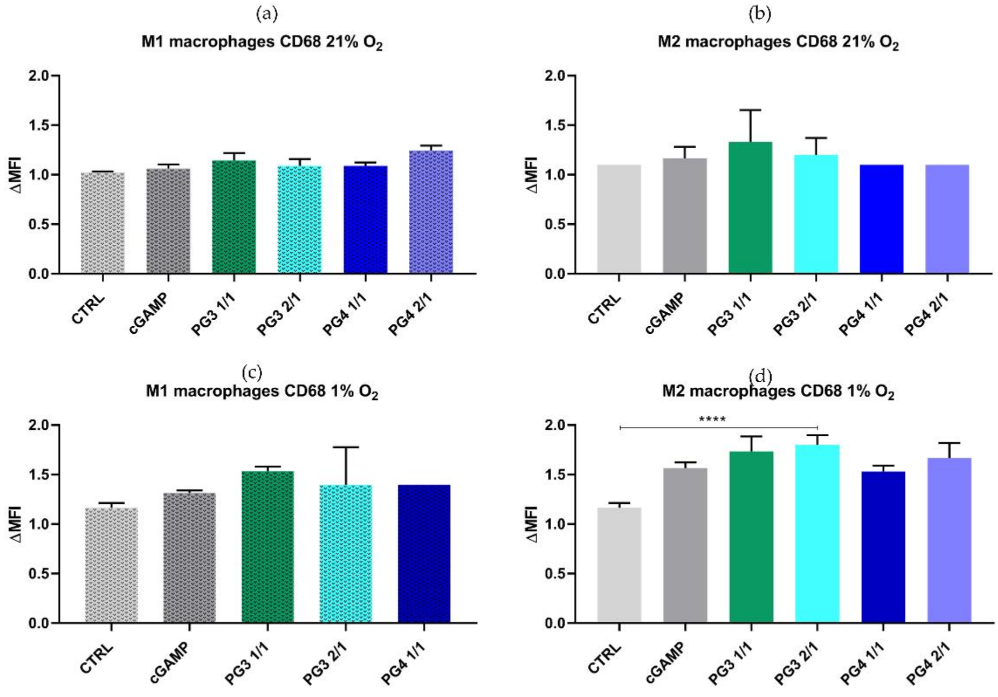

Figure S10. Surface expression of costimulatory molecule CD68 on murine BMDM cells. Polarized M1 and M2 BMDMs were stimulated in vitro with corresponding PAMAM NCs (PG3 1/1, PG3 2/1, PG4 1/1 or PG4 2/1), cGAMP (25 µg/condition) or with media under normoxic (**a**,**b**) or hypoxic (**c**,**d**) conditions for 24 h. The cells were stained with fluorophore-conjugated antibodies against the CD86 markers and analyzed by flow cytometry. Error bars are SD of three independent experiments. **** $p < 0.0001$.

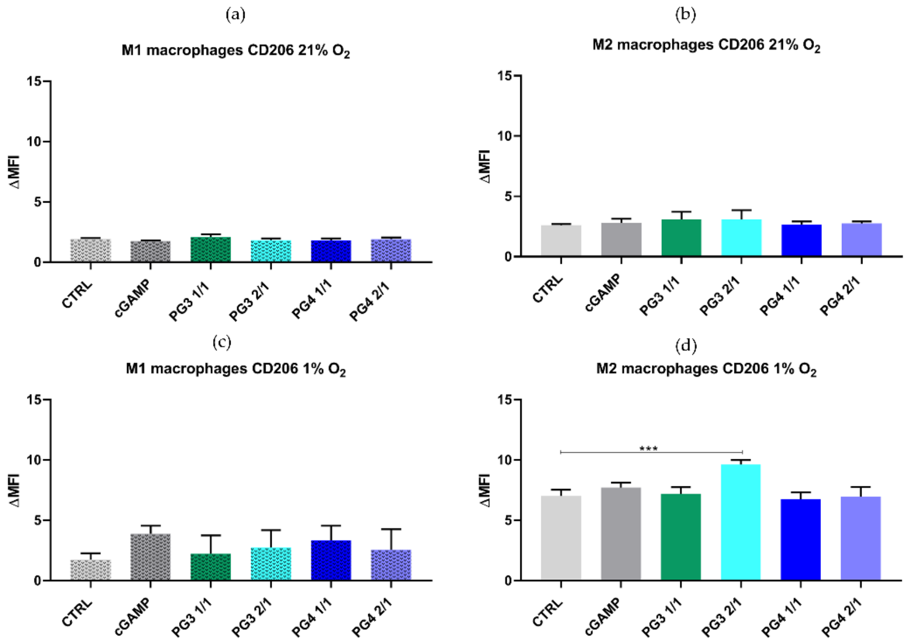

Figure S11. PAMAM up-regulates surface expression of costimulatory molecule CD80 on murine BMDM cells. Polarized M1 and M2 BMDMs were stimulated in vitro with corresponding PAMAM NCs (PG3 1/1, PG3 2/1, PG4 1/1 or PG4 2/1), cGAMP (25 µg/condition) or with media under normoxic (**a**,**b**) or hypoxic (**c**,**d**) conditions for 24 h. The cells were stained with fluorophore-conjugated antibodies against the CD86 markers and analyzed by flow cytometry. Error bars are SD of three independent experiments. *** $p < 0.001$.

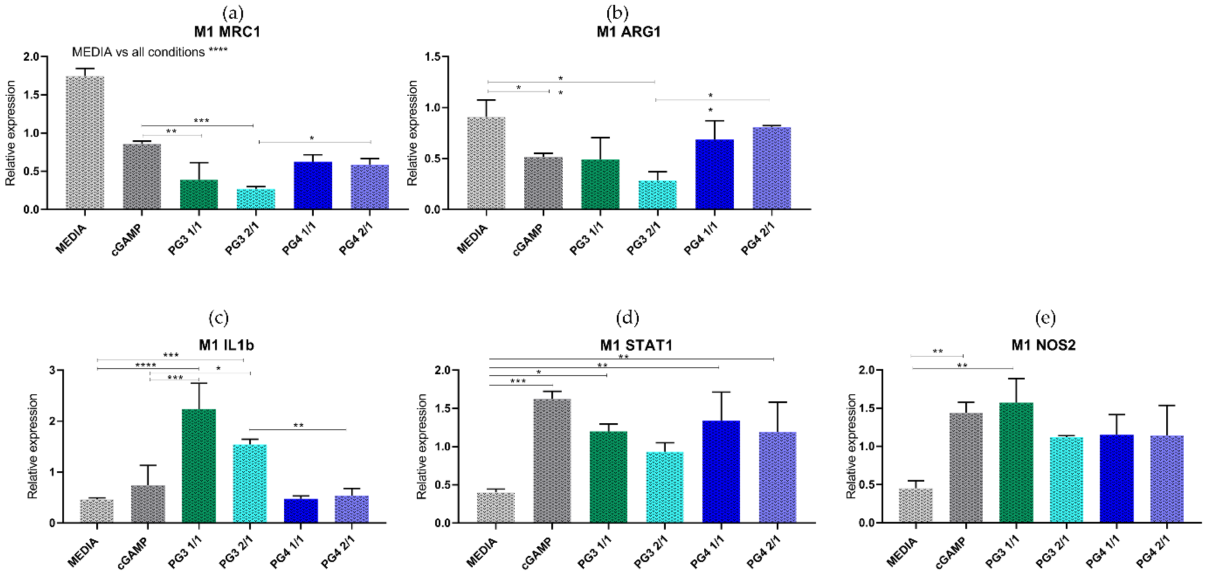

Figure S12. NC upregulates mRNA expression of pro-inflammatory (M1) genes and reduced the expression of anti-inflammatory (M2) genes in M1 polarized BMDMs. Relative mRNA expression of M2-like macrophage markers (Mrc1 (**a**) and Arg1 (**b**)) and of classically activated (M1-like) macrophage markers (IL-1b (**c**), STAT1 (**d**) and NOS2 (**e**)) in M2 BMDMs treated with corresponding PAMAM NCs(PG3 1/1, PG3 2/1, PG4 1/1 or PG4 2/1), cGAMP (25 µg/condition) or with media * $p < 0.05$; ** $p < 0.01$; *** $p < 0.001$; **** $p < 0.0001$.

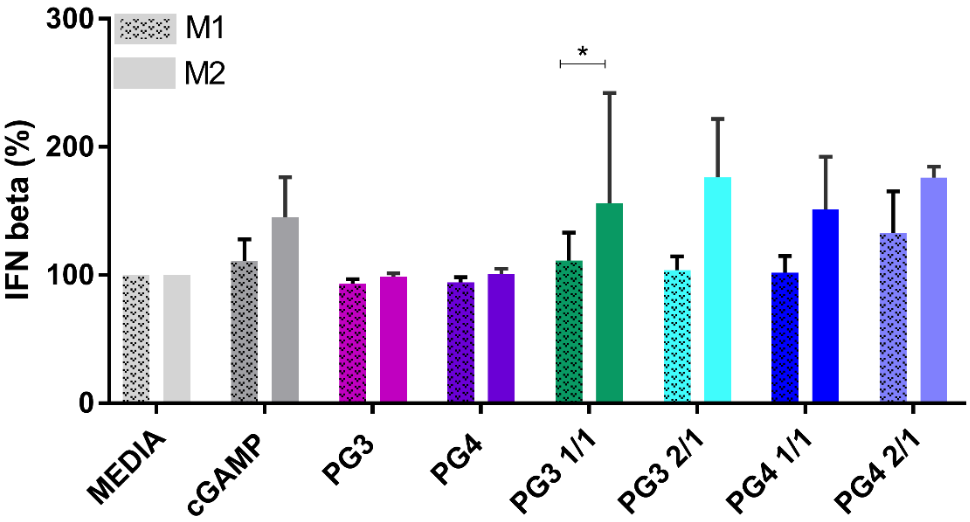

Figure S13. IFN beta level of M1 and M2 macrophages after 24h exposure of CTRL and NCs. * $p < 0.05$.
